# Diagnosis by Volatile Organic Compounds in Exhaled Breath from Lung Cancer Patients Using Support Vector Machine Algorithm

**DOI:** 10.3390/s17020287

**Published:** 2017-02-04

**Authors:** Yuichi Sakumura, Yutaro Koyama, Hiroaki Tokutake, Toyoaki Hida, Kazuo Sato, Toshio Itoh, Takafumi Akamatsu, Woosuck Shin

**Affiliations:** 1 Department of Information Science and Technology, Aichi Prefectural University, Nagakute 480-1198, Japan; sonic.h.0715@gmail.com (Y.K.); tokusanpc@gmail.com (H.T.); 2 Department of Thoracic Oncology, Aichi Cancer Center, 1-1 Kanokoden, Chikusa-ku, Nagoya 464-8681, Japan; 107974@aichi-cc.jp; 3 Department of Mechanical Engineering, Aichi Institute of Technology, Toyota, 470-0392, Japan; sato@aitech.ac.jp; 4 Department of Materials and Chemistry, National Institute of Advanced Industrial Science and Technology (AIST), Shimo-Shidami, Moriyama-ku, Nagoya 463-8560, Japan; itoh-toshio@aist.go.jp (T.I.); t-akamatsu@aist.go.jp (T.A.)

**Keywords:** lung cancer, volatile organic compounds (VOCs), exhaled air, screening, gas chromatography–mass spectrometry analysis, support vector machine (SVM)

## Abstract

Monitoring exhaled breath is a very attractive, noninvasive screening technique for early diagnosis of diseases, especially lung cancer. However, the technique provides insufficient accuracy because the exhaled air has many crucial volatile organic compounds (VOCs) at very low concentrations (ppb level). We analyzed the breath exhaled by lung cancer patients and healthy subjects (controls) using gas chromatography/mass spectrometry (GC/MS), and performed a subsequent statistical analysis to diagnose lung cancer based on the combination of multiple lung cancer-related VOCs. We detected 68 VOCs as marker species using GC/MS analysis. We reduced the number of VOCs and used support vector machine (SVM) algorithm to classify the samples. We observed that a combination of five VOCs (CHN, methanol, CH_3_CN, isoprene, 1-propanol) is sufficient for 89.0% screening accuracy, and hence, it can be used for the design and development of a desktop GC-sensor analysis system for lung cancer.

## 1. Introduction

Balancing the quality of life and sharp increase in healthcare expenses is an important social issue. Breath analysis is a noninvasive technique, allows easy sample collection, and provides quick results; thus, it is gaining attention as a new diagnostic technology. Breath is composed mainly of nitrogen (the most abundant gas in the atmosphere) along with carbon dioxide produced by respiration, oxygen that was not consumed, and water vapor. In addition, it contains more than 100 additional types of gas components in different concentrations, which provide information that may be useful to monitor health conditions such as disease or stress. Gas-sensing technologies (e.g., selective and quantitative gas detection) are necessary to measure the concentration of different gas species related to halitosis, metabolism, and diseases.

Some volatile organic compounds (VOCs) in exhaled breath are expected to be useful as biomarkers for diseases, including cancer [1,2]. Lung cancer has become a major concern in Japan because it is the top cause of death by disease in the country. Lung cancer has a high mortality rate because it has often progressed by the time a patient perceives any symptoms and is diagnosed. If lung cancer is detected earlier, it can be treated by surgery and subsequent chemotherapy. However, no good early diagnostics are available. It is difficult to detect lung cancer using chest X-ray radiography (CXR) [3]; thus, diagnosis is provided after sputum analysis and extensive examination with low-dose computed tomographic (LDCT) scanning.

Monitoring the breath is one of the most noninvasive screening techniques available for early diagnosis [4,5,6,7,8,9,10,11]; however, this method is limited by its poor accuracy because the exhaled breath has many VOCs at very low concentrations (ppb level), and there is no clear protocol of breath sampling [12]. Gas chromatography–mass spectrometry (GC/MS) is one of the best methods for detecting low-concentration VOCs; however, this method is expensive, and the instrumentation is not portable.

If some specific VOCs have sufficient information for diagnosing diseases, one could measure these VOCs with relatively high resolution using a suitable technique, and detection of the other gas species would not be necessary. VOCs have been reported as biomarkers for lung cancer [5,8,13,14,15,16,17,18]; however, various other compounds have also been reported as possible biomarkers, suggesting disagreement in literature. For these reasons, VOC patterns (i.e., not single VOCs, but combinations of several VOCs) should be used for exhaled breath analysis for the diagnosis of diseases [5,8,13,14,15,16,17,18,19]. Here, we seek to determine which gas species are more important and how many are necessary to ensure system reliability. The optimized prototype system should be of reasonable size and cost; the number of gas species used should be fewer than 10, and the system should be able to detect the essential components of those gases.

Recent studies have demonstrated computer-assisted diagnosis by measuring multiple VOCs. Various algorithms have been applied to examine lung cancer diagnosis using multiple VOCs; for example, forward stepwise discriminant analysis [5,13], partial least-squares regression [14], logistic regression [15,18], random forest classification [20], weighted digital sum discriminator [21], and linear canonical discriminant analysis with principal component analysis (PCA) [17]. The support vector machine (SVM) is a powerful supervised machine learning model based on statistical learning theory [22]. SVM has been successfully used in the field of brain science to classify brain tumors [23,24], Alzheimer’s disease [25,26], and depression [27,28], based on magnetic resonance imaging (MRI) data. SVM-based research has been performed for VOC analysis to classify lung cancer cells [29], smoking subjects [30], patients with chronic obstructive pulmonary disease [31], and patients with head-and-neck cancer [32]. Many studies have examined VOCs in the exhaled breath; however, SVM-based analysis with a raw VOC data set has not been examined to select the essential VOCs for diagnosing lung cancer.

We have developed a prototype system for monitoring exhaled breath that can replace GC/MS. It combines a highly sensitive gas sensor with GC to separate the gases. The prototype system has simple GC columns, a simple gas-condenser unit, and SnO_2_-based semiconductor gas sensors [33]. For further development of the prototype system, we analyzed the VOCs detected in the breath of lung cancer patients and healthy subjects (controls) to determine the most effective combination of VOCs for diagnosing lung cancer. We applied a nonlinear SVM classification to various subsets of the VOCs detected by the GC/MS system and did not preprocess the VOCs by PCA because it is difficult to select VOCs from the principal components, which are composed of the multiple VOC features, and validate diagnose ability by the selected VOCs. We also did not select VOCs that have a significant difference in concentration between cancer and healthy samples. It is likely that the diagnosis is possible by the VOCs that have no significant difference in VOC concentration. We performed leave-one-out cross-validation of the samples (patients and controls) for each of the combinations of VOCs and evaluated the true positive rate (sensitivity) and accuracy of diagnosis for the left-out sample. We found that a specific combination of a small number of VOCs could diagnose lung cancer with a high accuracy.

## 2. Breath Gas Analysis and Diagnosis Methods 

### 2.1. Breath Collection and Analysis

After obtaining approval from the local ethics committees of Aichi Cancer Center and the National Institute of Advanced Industrial Science and Technology and written informed consent from the participants, 107 patients with lung cancer and 29 healthy individuals were enrolled in this study. The stage and histology of the lung cancer were omitted and it was simply labeled as “lung cancer”. The numbers of patients with stage I, II, III and IV lung cancer were 55, 15, 28 and 9, respectively. The numbers of smoker, ex-smoker, and nonsmoker for lung cancer patients were 47, 15 and 45, and those for healthy individuals were 5, 3 and 21, respectively.

**Figure 1:**
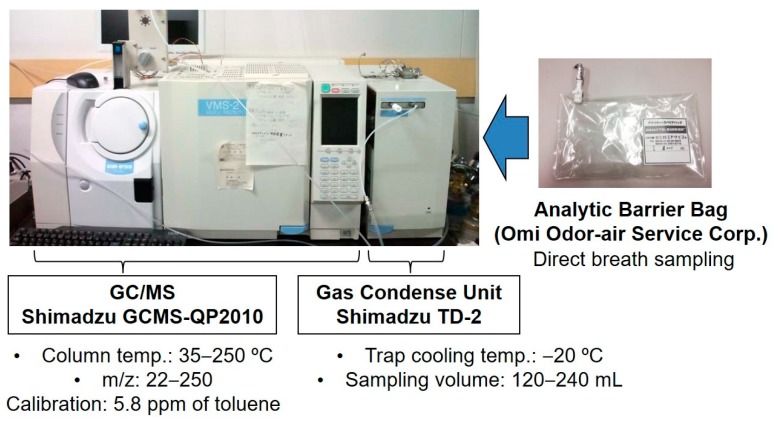
Breath sampling and gas analysis by GC/MS.

Human breath and ambient air in a room at Aichi Cancer Center were collected using an Analytic Barrier Bag (Omi Odor-Air Service Corp., Omihachiman, Japan). Before sample collection, the volunteers did not eat or smoke for several hours and they stayed in the room for at least 10 min. All volunteers blew their alveolar breath into a 1 L Analytic Barrier Bag immediately after they exhaled their respiratory tract air in a consultation room. The breath was analyzed using a GCMS-QP2010 instrument (Shimadzu, Kyoto, Japan) equipped with a TD-2 gas-condensing unit (Shimadzu) (Figure 1). The TD-2 has a gas aspiration unit and a cold trap for condensation of low-concentration VOCs. The GC/MS system used helium gas (99.9995% purity, Taiyo Nippon Sanso, Japan) as the carrier gas. A DB-1 series 123-1063 gas column (Agilent Technologies, Santa Clara, CA, USA) was used. The background VOCs in the room air and the VOCs from the exhaled air were analyzed, and the concentrations of background VOCs were subtracted from the results for the exhaled air prior to data analysis. The concentrations of these VOCs were excluded from the results of breath analysis in this study. The details are reported elsewhere [33].

We detected 63 VOCs. Among them, three VOCs may come from cancer treatment drugs and 40 VOCs were present at low concentrations, close to the detection limit of GC/MS. We deleted these 43 VOCs and examined the SVM diagnosis using the remaining 20 VOCs (Table 1).

### 2.2. Data Sets

**Figure 2:**
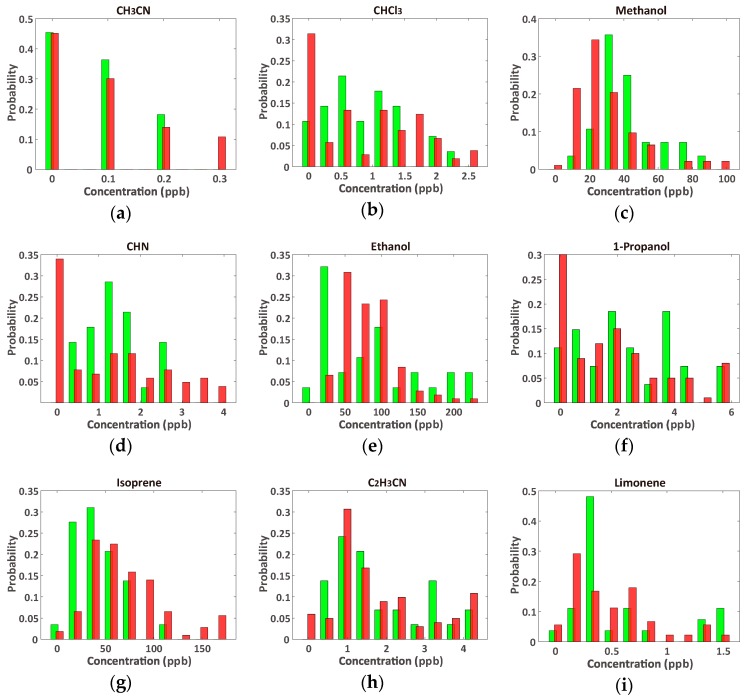
Comparison of VOC concentration distributions from lung cancer (red, *n* = 107) and healthy (green, *n* = 29) controls’ breath; (**a**) CH_3_CN; (**b**) CHCl_3_; (**c**) methanol; (**d**) CHN; (**e**) ethanol; (**f**) 1-propanol; (**g**) isoprene; (**h**) C_2_H_3_CN; and (**i**) limonene. The VOCs in (**a**–**e**) show significant differences between samples, while those in (**f**–**i**) do not show significant differences (Table 1). The distributions of the remaining 11 VOCs are shown in the Supplementary Information (Figure S1).

As listed in Table 1, many of the VOCs have no significant differences between cancer and healthy samples, and the concentration distributions of the VOCs (nine VOCs are selected and shown in Figure 2) show unclear boundaries between the cancer and healthy control samples. Some of the lung cancer samples contained higher concentrations of VOCs than the healthy samples; however, there was a wide overlap of the types of VOCs found in each sample. This suggests that it is quite difficult to diagnose lung cancer using a single VOC; thus, diagnosis using multiple VOCs is necessary.

There are 1,048,575 (=∑i=120Ci20, where Ckn represents k-combinations of n elements) VOC combinations of the 20 VOCs listed in Table 1. We applied nonlinear SVM diagnosis to each of the combinations and evaluated their accuracy levels, as described below. A VOC that has no contribution toward improving the diagnostic accuracy should be removed from the data set even if it has a large contribution to the principal component space, and vice versa. In addition, reducing the number of possible VOCs is helpful for designing a portable VOC detector.

**Figure 3:**
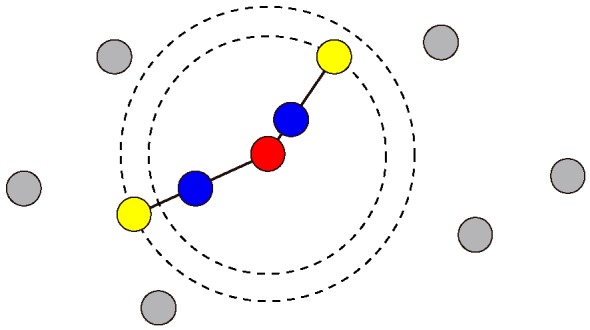
Schematic illustrating the oversampling technique to obtain the same number of healthy control samples to that of the lung cancer patients. After one sample (**red**) is randomly chosen, two samples (**blue**) are randomly interpolated on the lines between the chosen sample and the two nearest samples (**yellow**).

The imbalanced sample numbers in the VOC data set (lung cancer patients: 107; healthy individuals: 29) likely cause an inappropriate classification. To complete the sample numbers, we introduced a synthetic minority oversampling technique [34,35,36]. One original sample (healthy control in our case) was randomly chosen, and two virtual samples were interpolated at a random point between the chosen sample and the two samples that are nearest to the chosen one (case of nearest number k=2; Figure 3). By repeating this process, we provided 107 healthy control samples.

### 2.3. SVM Classifier

**Figure 4:**
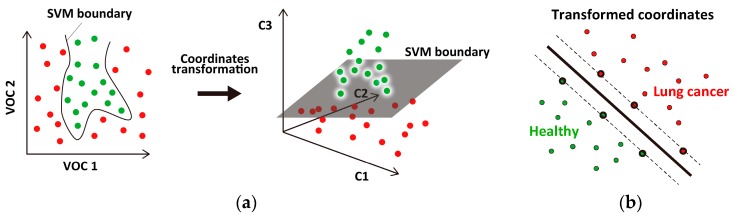
Schematic illustrating nonlinear support vector machine (SVM). (**a**) The two-class data set is composed of two VOCs (VOC 1 and VOC 2; left panel), which are transformed into a different coordinate space (right panel) where the dataset can be classified by a flat boundary; (**b**) The SVM boundary (thick line) is determined using data points called support vectors (thick circles). The number of support vectors should be small to avoid overfitting the data points.

SVM is an algorithm that determines a flat classification boundary between two-class data sets. The concentration distribution of the selected VOCs is broad and shows unclear boundaries between the cancer and healthy samples; thus, it is difficult to clearly classify the VOC samples using linear SVM [22]. We introduced a nonlinear SVM [37] with a Gaussian kernel function, which is widely used for classifying biological data sets (e.g., microarray gene expressions [38,39,40], DNA fragments [41], and cell shapes [42]). In general, the data point coordinates are transformed to a higher dimensional coordinate space, where SVM can draw a flat boundary between the transformed two-class data sets (Figure 4). The coordinate transformation is characterized by the kernel function. We used a Gaussian kernel function, $\exp\left(-{∥x_{1}-x_{2}∥}^{2}/2\sigma^{2}\right)$, where $x_{1}$ and $x_{2}$ represent normalized VOC data points and σ is a parameter that scales the distance between the points. Another parameter, C, regulates the penalty for misclassification. Here, we set σ=1.5 and C=1000 to reduce the number of data points that determine the classification boundary (support vectors), by which SVM can avoid overfitting the dataset. All computations were performed using SVM functions (svmtrain, svmclassify) within the Statistics and Machine Learning Toolbox of MATLAB (MathWorks).

### 2.4. Evaluation of Classification Accuracy

**Figure 5:**
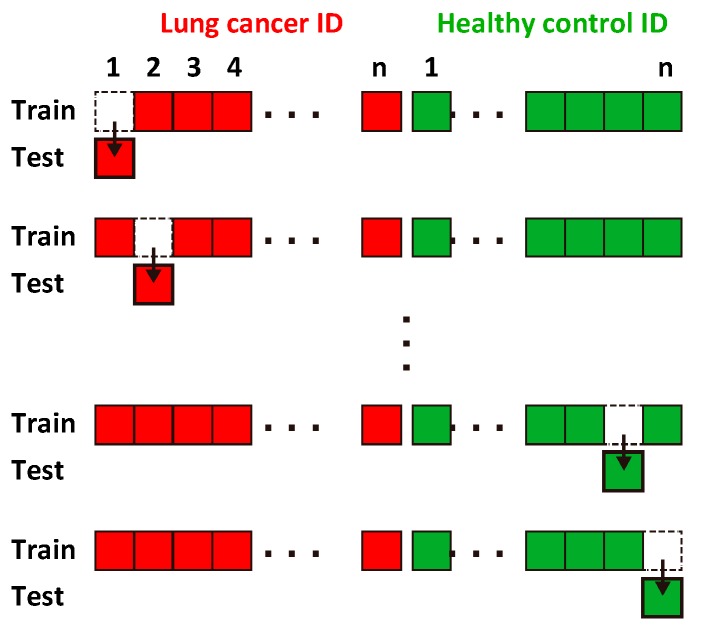
Schematic illustrating the leave-one-out cross-validation (LOOCV) procedure. A data point is repeatedly exchanged to categorize the training and testing data set.

We introduced the leave-one-out cross-validation (LOOCV) method, which is widely used in biology [43,44] and breath gas analysis [5,14,45,46], to evaluate the capability of the SVM diagnosis for the given data set. In LOOCV, one data point is left out of the data set to evaluate the accuracy of the diagnosis, while the remaining data points are used to train the classifier. Then, the left-out data point is diagnosed by the trained classifier (Figure 5). This process is repeated for each sample to compute the true positive rate (TPR =TP/(TP+FN)), true negative rate (TNR =TN/TN+FP), and accuracy (ACC =(TP+TN)/(TP+FN+TN+FP)), where 29 healthy controls were used as true negative samples. These values equal 100% if a completely accurate diagnosis is achieved. We applied LOOCV to all of the VOC combinations to screen the effective combinations for cancer diagnosis.

## 3. Results and Discussion 

### 3.1. Optimal Number of VOCs for Classification

**Figure 6:**
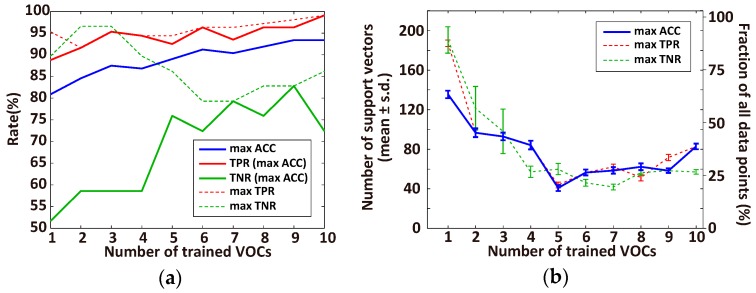
Dependency of the performance of SVM diagnosis on the number of trained VOCs of the data set (lung cancer patients, *n* = 107; healthy individuals, *n* = 29, oversampling healthy samples, *n* = 78). (**a**) Best accuracy (ACC, blue line) with the corresponding true positive rate (TPR, solid red line) and true negative rate (TNR, solid green line) within all combinations of each number of trained VOCs (from 1 to 10). The dashed red and green lines represent the best TPR and TNR, respectively; (**b**) The number of support vectors that are used in the classifier in (**a**) for the best ACC (blue), TPR (red), and TNR (green). Left and right y-axes represent the actual number of data points and fraction of all data points, respectively.

The diagnostic accuracy using the original data set (*n* = 107 for lung cancer patients and *n* = 29 for healthy individuals) and oversampled healthy samples (*n* = 78) depends on the number of VOCs trained by the SVM classifier, as summarized in Figure 6a. The accuracy increases as more VOCs are included, and the maximum accuracy is achieved using 9 or 10 VOCs, while the best TPR is saturated even for one VOC, and the best TNR decreases above 4 VOCs. In contrast, the numbers of corresponding support vectors of the ACC and TPR classification are the lowest (18.7% of the data points) when there are 5 trained VOCs, and that of TNR reaches almost bottom for 4 VOCs (Figure 6b). These results suggest that, without overfitting, 5 VOCs are sufficient for 89.0% diagnostic accuracy, and that the 95% TPR- and 89% TNR-based diagnoses are possible when using 5 and 4 VOCs, respectively.

### 3.2. Effective VOC Combinations for Diagnosing Lung Cancer

We determined effective VOC combinations for diagnosing lung cancer with high values for ACC (Table 2), TPR (Table 3), and TNR (Table 4) by fixing the number of trained VOCs at the value that provided a small number of support vectors (*n* = 5 for ACC, *n* = 5 for TPR, and *n* = 4 for TNR; Figure 6b). The VOC combinations are sorted by ACC, TPR, and TNR. The most important VOC combinations could not be determined because the differences between the rates in these tables are not large, and the VOC concentrations may contain noises caused by the sensor or sampling process. However, certain VOCs were common in each combination while some VOCs (e.g., nonanal and toluene) were rarely used for diagnosis.

The variation of VOC combinations in the top diagnosis above was probably caused by the noise in the detected concentration, cancer type, or cancer stage. We extracted the VOCs that were frequently present in the top 10 combinations (Table 5). The results show that: (1) CH_3_CN and isoprene are commonly used for all diagnoses; (2) the frequently used combination in ACC is the same as the best combination in Table 2; (3) 1-propanol, C_2_H_3_CN, and ethanol are specific to the TPR-based diagnosis; (4) the frequently used combination in TPR is same as the third combination in Table 3; (5) CHN, CH_3_CN, and methanol are specific to the TNR-based diagnosis; (6) the frequently used combination in TNR, except for methanol, is the same as the second combination in Table 5; and (7) the group of VOCs in the ACC-based diagnosis (“Top ACC” in Table 5) contains a mixture of VOCs from the TPR- and TNR-based diagnoses and justifies the definition of ACC (i.e., the indicator merging TPR and TNR). If the eight VOCs listed in Table 5 (two from ACC, three from TPR, and three from TNR) were used, the SVM diagnosis would show a performance of 84.6% for ACC, 91.6% for TPR, and 58.6% for TNR, with 54.2 ± 5.47 for the number of support vectors. This is not a particularly bad performance, but TNR, in particular, would provide a low performance. This is probably caused by the small number of original healthy controls. The extracted VOCs from the VOCs in Table 1 are different from the result of our previous study [33], where the selection of target VOCs is different.

**Figure 7:**
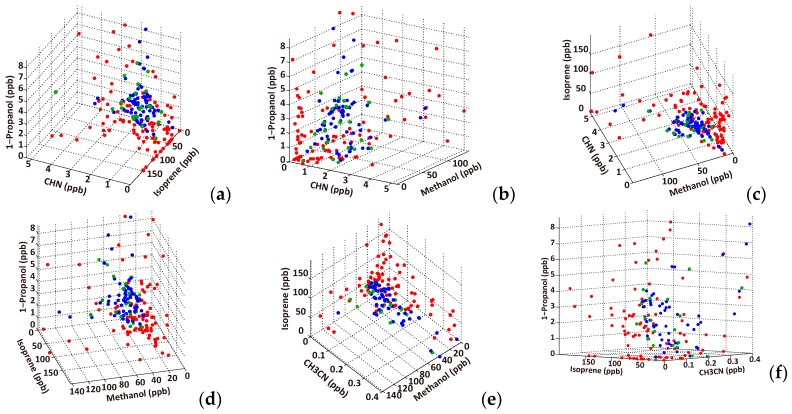
VOC distributions on a 3D representation for the list of top accuracy combinations in Table 5. CHN, isoprene, 1-propanol (**a**); CHN, methanol, 1-propanol (**b**); CHN, methanol, isoprene (**c**); isoprene, methanol, 1-propanol (**d**); CH_3_CN, methanol, isoprene (**e**); and isoprene, CH_3_CN, 1-propanol (**f**). The red and green circles represent lung cancer patients and healthy controls, respectively, and the blue circles indicate the oversampling data. The oversampling data are more widely spread than the original healthy samples in this range because some of the healthy samples exist outside of the axis range.

Furthermore, to examine the discriminability between the cancer and healthy samples, the scatter diagrams for six combinations of three VOCs in Table 5 were plotted on 3D coordinates (Figure 7). The results represent much smaller overlaps between the lung cancer and healthy groups than the 1D representation in Figure 2, and suggest a high discriminability between cancer patients and healthy subjects using the VOC combination rather than single VOCs.

### 3.3. Correlation between Cancer Stage and Distance from the Classification Boundary

**Figure 8:**
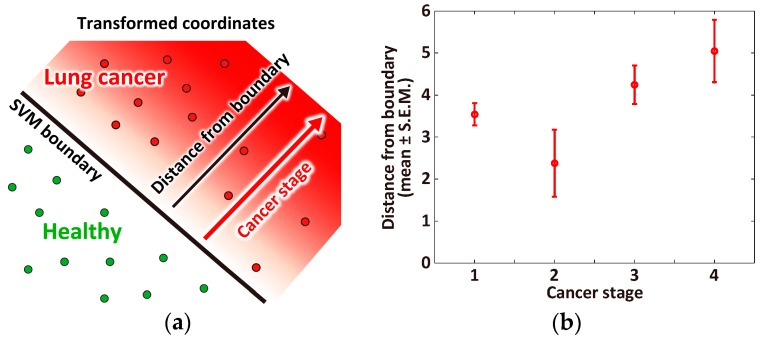
(**a**) Schematic illustration of the hypothesis that the cancer stage correlates with distance from the SVM boundary in the transformed coordinates space; (**b**) The *y*-axis indicates the distance from the SVM boundary. The learning VOC combination of the best TPR in Table 3 (butane, ethanol, acetone, C_2_H_3_CN, and toluene) was used for computing the test sample distance.

Data points near the classification boundary contain a property of each class because SVM provides a boundary between the two-class data points. In other words, the data points that are far from the boundary have the specific property of their class. In the SVM diagnosis, the data samples of low cancer stages are located near the boundary and those of high stages are far from the boundary (Figure 8a). Such a distance-based feature extraction has been theoretically studied [47,48] and applied to MRI images of the brain [49]. Thus, we computed the distances of the cancer samples from the boundary using the best VOC combination in the TPR rank with the LOO fashion; the test sample distances are computed by the classifier developed by the remaining learning samples. The higher cancer stage samples are located relatively far from the boundary (Figure 8b). This suggests that the SVM diagnosis could be used for estimating the cancer stage of a patient. The first-stage patients have relatively long distances. This may be caused by noise in VOCs, mislabeling of stage, or nonlinear transformation of the VOCs near the boundary.

## 4. Summary and Conclusions

We have applied nonlinear SVM classification to the detection of VOCs for lung cancer diagnosis with leave-one-out cross-validation, and have determined the optimal VOC patterns for the diagnosis. Optimal combinations of VOCs depend on ACC, TPR, or TNR (Table 2, Table 3 and Table 4). The TPR- and TNR-based optimal combinations are useful for biologically investigating why cancer patients and healthy people are characterized by these VOCs. The ACC-based optimal combinations will be used for diagnosing subjects. The TPR-based diagnosis is better for avoiding a risk of false negative. The efficient strategy is to develop a diagnosis tool based on the ACC-based diagnosis while the TPR-based diagnoses is used in hospitals because improving the ACC diagnosis also improves the TPR diagnosis.

The TNR results were lower than that for TPR for all VOC combinations. This is possibly caused by the oversampling of the healthy controls and will be improved by collecting VOCs from more healthy individuals. For the correlation between the SVM distance and cancer stage, a possible alternative application would be to classify samples into 5 classes (stages 1–4 and healthy) by SVM. This work may be performed in the future, because we cannot currently obtain a high accuracy using a multiclass SVM.

The optimal VOC set was selected based on the VOC concentrations, each of which was detected by the same GC/MS. There is little quantitative variation added by the GC/MS. If we use a different GC/MS that has different VOC sensitivities, some VOCs will have different measured concentrations. Even in this case, the SVM diagnosis will select effective VOC combinations similar to those in this work, because the VOC concentrations are normalized in the SVM; a relative concentration correlation between samples is important for classification. However, this argument does not hold, and different VOC combinations are possibly selected, in the case that the detected VOCs differ depending on the GC/MS because of VOC sensitivity.

We showed that a diagnosis with 89.0% accuracy can be performed using five VOCs. This highly efficient SVM classification will be integrated into the prototype breath analyzer for lung cancer screening utilizing double GC columns and sensors [33], and a new breath test in Aichi Cancer Center is going to start. It must be confirmed that the selected VOCs are also optimal sets when we diagnose with the prototype analyzer specialized to these optimal VOCs in future.

We promote this integration and prototype analyzer, expecting that the SVM classifier can be used for the further development of a desktop GC-sensor analysis system for lung cancer. Furthermore, if the precise VOC composition of five VOC mixtures is measured, the cancer stage can be predicted, as it is correlated with the distance of a cancer sample from the SVM classification boundary.

## Figures and Tables

**Table 1:** Selected volatile organic compounds (VOCs) for the computer-assisted diagnostic analysis.

Butane ^†,‡^	CH_3_CN ^†,‡^	CHCl_3_^†,‡^	Methanol ^†^	Acetone ^‡^
CHN ^‡^	Ethanol ^‡^	1-Propanol	2-Propanol	C_8_H_16_
Isoprene	Dichlorobenzene	C_8_H_17_OH	Xylene	Methylcyclohexane
Toluene	C_2_H_3_CN	Limonene	Nonanal	Unknown ^1^

^†^ Wilcoxon test: p<0.05; ^‡^ Kolmogorov–Smirnov test: p<0.05; ^1^ this VOC could not be identified.

**Table 2:** Top 10 VOC combinations sorted by ACC (%) (5 VOCs were trained, MC = methylcyclohexane; boldface: most frequent VOC).

Rank	1	2	2	2	2	6	6	6	9	9
**ACC**	**89.0**	**88.2**	**88.2**	**88.2**	**88.2**	**86.8**	**86.8**	**86.8**	**86.0**	**86.0**
TPR	92.5	91.6	93.5	91.6	92.5	91.6	89.7	92.5	91.6	87.9
TNR	75.9	75.9	69.0	75.9	72.4	69.0	75.9	65.5	65.5	79.3
VOCs	CHN	CHN	Methanol	Methanol	Butane	CHN	CHN	C_2_H_3_CN	CHN	CHN
	Methanol	CH_3_CN	Acetone	Isoprene	CH_3_CN	Methanol	Butane	**Isoprene**	Ethanol	CH_3_CN
	CH_3_CN	C_2_H_3_CN	C_2_H_3_CN	Xylene	**Isoprene**	CH_3_CN	CH_3_CN	1-Propanol	**Isoprene**	**Isoprene**
	**Isoprene**	**Isoprene**	**Isoprene**	Unknown-1	1-Propanol	1-Propanol	**Isoprene**	Unknown-1	1-Propanol	CHCl_3_
	1-Propanol	CHCl_3_	1-Propanol	C_8_H_17_OH	Xylene	MC	CHCl_3_	C_8_H_17_OH	Toluene	Xylene

**Table 3:** Top 10 VOC combinations sorted by TPR (%) (5 VOCs were trained, MC = methylcyclohexane; boldface: most frequent VOC).

Rank	1	2	3	3	3	3	3	3	3	10
ACC	84.6	88.2	86.0	89.0	84.6	88.2	85.3	85.3	86.8	86.8
**TPR**	**94.4**	**93.5**	**92.5**	**92.5**	**92.5**	**92.5**	**92.5**	**92.5**	**92.5**	**91.6**
TNR	48.3	69.0	62.1	75.9	55.2	72.4	58.6	58.6	65.5	69.0
VOCs	Butane	Methanol	Ethanol	CHN	CHN	Butane	Ethanol	Ethanol	C_2_H_3_CN	CHN
	Ethanol	Acetone	CH_3_CN	Methanol	Ethanol	CH_3_CN	CH_3_CN	CH_3_CN	Isoprene	Methanol
	Acetone	C_2_H_3_CN	C_2_H_3_CN	CH_3_CN	C_2_H_3_CN	Isoprene	Acetone	MC	**1-Propanol**	CH_3_CN
	C_2_H_3_CN	Isoprene	Isoprene	Isoprene	**1-Propanol**	**1-Propanol**	2-Propanol	Unknown-1	Unknown-1	**1-Propanol**
	Toluene	**1-Propanol**	**1-Propanol**	**1-Propanol**	CHCl_3_	Xylene	C_2_H_3_CN	C_8_H_17_OH	C_8_H_17_OH	MC

**Table 4:** Top 10 VOC combinations sorted by TNR (%) (4 VOCs were trained, MC = methylcyclohexane; boldface: most frequent VOCs).

Rank	1	2	2	2	5	5	5	5	5	10
ACC	84.6	89.0	88.2	84.6	88.2	85.3	86.0	85.3	86.8	86.8
TPR	82.2	86.9	76.6	78.5	77.6	79.4	72.9	78.5	71.0	83.2
**TNR**	**89.7**	**86.2**	**86.2**	**86.2**	**82.8**	**82.8**	**82.8**	**82.8**	**82.8**	**79.3**
VOCs	**CHN**	**CHN**	**CHN**	**CHN**	**CHN**	**CHN**	Methanol	**CH_3_CN**	**CH_3_CN**	**CHN**
	Isoprene	**CH_3_CN**	Methanol	**CH_3_CN**	Methanol	Methanol	**CH_3_CN**	Acetone	Isoprene	Methanol
	Xylene	Isoprene	2-Propanol	CHCl_3_	**CH_3_CN**	Isoprene	Isoprene	Unknown-1	MC	**CH_3_CN**
	Limonene	CHCl_3_	Nonanal	Dichlorobenzene	C_2_H_3_CN	Limonene	Limonene	C_8_H_17_OH	Nonanal	CHCl_3_

**Table 5:** VOCs that are frequently used in the top 10 ACC (Table 2), TPR (Table 3), and TNR (Table 4) combinations. The VOCs written in boldface are the same as those in Table 2, Table 3 and Table 4 and represent the VOCs that are used most frequently in the ACC-, TPR-, or TNR-based diagnoses. VOCs commonly used in every diagnosis and those specifically used for TPR- and TNR-based diagnoses are colored by blue, red, and green, respectively.

ACC			TPR			TNR		
Rank	VOC	Count	Rank	VOC	Count	Rank	VOC	Count
1	**Isoprene**	9	1	**1-Propanol**	7	1	**CHN**	7
2	CHN	6	2	C_2_H_3_CN	6	1	**CH_3_CN**	7
2	1-Propanol	6	2	CH_3_CN	6	3	Methanol	5
2	CH_3_CN	6	4	Ethanol	5	3	Isoprene	5
5	Methanol	4	4	Isoprene	5	5	CHCl_3_	3

## Supplementary Materials

The following are available online at http://www.mdpi.com/1424-8220/17/2/287/s1, Figure S1: VOC concentration distributions from lung cancer (red, *n* = 107) and healthy (green, *n* = 29) controls’ breath.
