# Supplementary material for: Diagnosis by Volatile Organic Compounds in Exhaled Breath from Lung Cancer Patients Using Support Vector Machine Algorithm
Source: Sensors (Basel). 2017 Feb 4;17(2):287. doi: 10.3390/s17020287 (PMC5335963; doi:10.3390/s17020287)
Supplement: Supplementary file 1 [file sensors-17-00287-s001.pdf]

Supplementary Materials: Diagnosis by Volatile Organic Compounds in Exhaled Breath from Lung Cancer Patients Using Support Vector Machine Algorithm

Yuichi Sakumura, Yutaro Koyama, Hiroaki Tokutake, Toyoaki Hida, Kazuo Sato, Toshio Itoh, Takafumi Akamatsu and Woosuck Shin

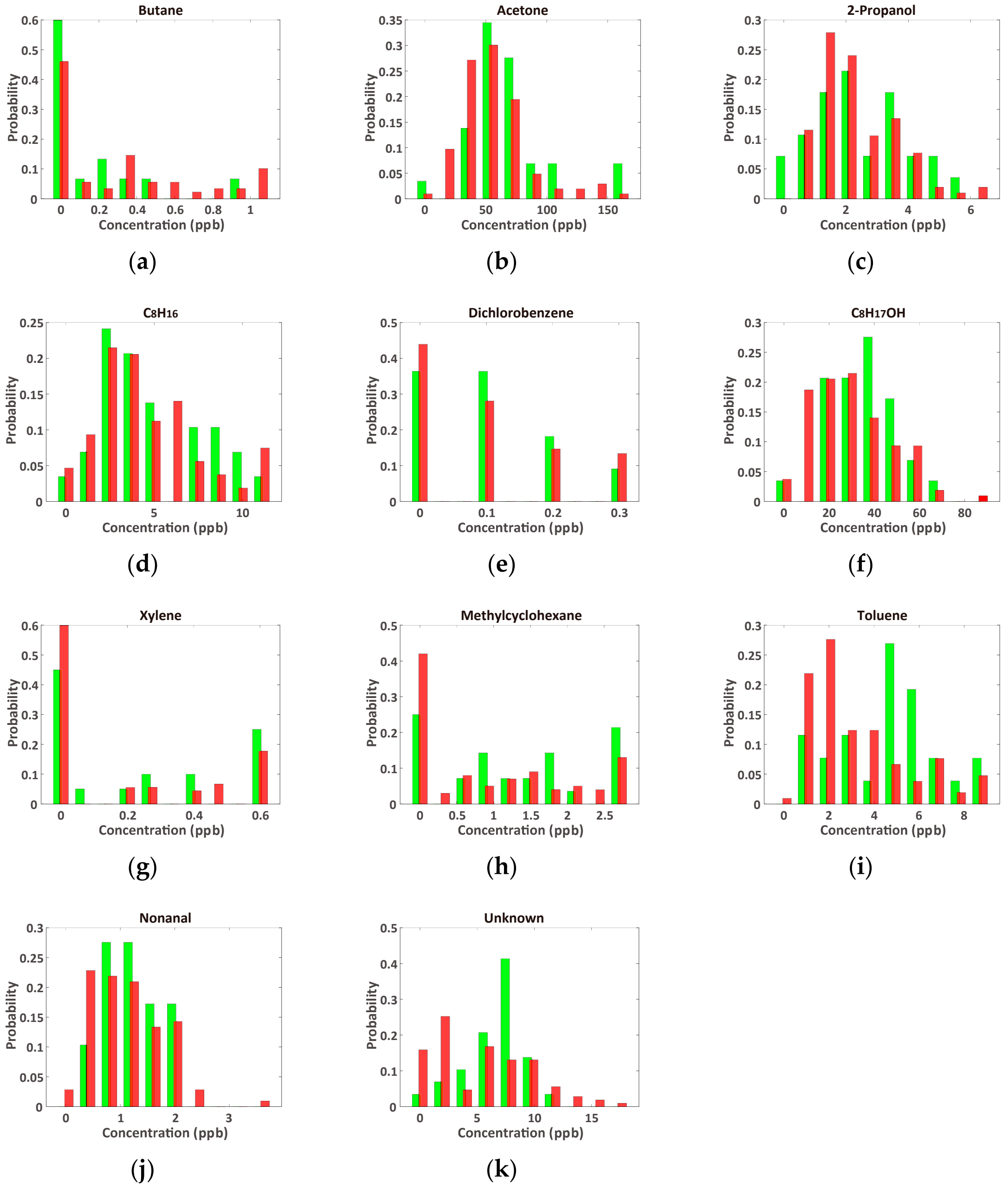

Figure S1. VOC concentration distributions from lung cancer (red, n = 107) and healthy (green, n = 29) controls' breath; (**a**) butane; (**b**) acetone; (**c**) 2-propanol; (**d**) C_8H_{16}; (**e**) dichlorobenzene; (**f**) $C_8H_{17}OH$; (**g**) xylene; (**h**) methylcyclohexane; (**i**) tolunene; (**j**) nonanal; and (**k**) unknown compound.
